# Language and Reading: the Role of Morpheme and Phoneme Awareness

**DOI:** 10.1007/s40474-018-0153-2

**Published:** 2018-10-31

**Authors:** Lynne G. Duncan

**Affiliations:** Psychology, University of Dundee, Dundee, DD1 4HN UK

**Keywords:** Reading development, Phoneme awareness, Morpheme awareness, Developmental dyslexia, Meta-linguistic awareness, Cross-linguistic

## Abstract

**Purpose of Review:**

Although spoken language in the form of meta-linguistic awareness is widely regarded as being involved in reading development, the extensive literature based on different experimental tasks, age groups, and languages makes it difficult to establish consensus about the type of awareness that is critical and the mechanisms underlying this relationship. The purpose of this review is to explore the links between reading and two specific aspects of meta-linguistic awareness, namely, phoneme awareness and morpheme awareness.

**Recent Findings:**

Research has uncovered distinct levels of meta-linguistic awareness that stand in different relationships to learning to read. Empirical findings support the reciprocal involvement of an awareness of phonemes and morphemes in reading development but the precise nature of the relationship between spoken and written language is subject to cross-language variation.

**Summary:**

A universal model of reading development is needed that is sufficiently flexible to allow interplay in the processing of phonology, orthography, and meaning in response to the linguistic characteristics of the spoken and written forms of the language being acquired. The linguistic characteristics that influence the development of phoneme and morpheme awareness are compared for alphabetic and morphographic orthographies and related to typical and atypical patterns of reading acquisition.

## Introduction

The words in our spoken languages can be broken down into smaller components known as phonemes (units of sound) and morphemes (units of meaning). These are more formally defined in the following: (a) phonemes are the smallest unit of sound to make a meaningful difference to a word; for example, the word cat contains three phonemes /k/-/a/-/t/; (b) morphemes are the basic units of meaning within words; for example, a free morpheme like cat is a word in its own right but bound morphemes like affixes (e.g. -er, -ing, un-) occur only in combination with a base (e.g. cooker).

Awareness of each of these units is a more specific form of general meta-linguistic awareness, which is the ability to reflect on language in contrast to the more direct usage of language for everyday communication and understanding. Even preschoolers with typical language development can still require time before demonstrating meta-linguistic awareness, possibly due to its dependence on the development of more domain-general skills such as decentration [1] or executive functions (e.g. cognitive flexibility [2]). The other important factor is educational input about literacy itself, since illiterate adults show little awareness of linguistic units such as phonemes [3••].

One theory of meta-linguistic development to have formalised these observations proposes the following developmental sequence [4]. Linguistic information is initially represented *implicitly*. This is described as an obligatory phase in typical spoken language development, which is sufficient to produce accurate behavioural performance but has limited flexibility to generalise to other situations. As children become more *explicitly* or consciously aware of linguistic information, meta-linguistic control is evident in their ability to manipulate this information in a variety of linguistic awareness tasks. Nevertheless, this is an optional phase, which requires the presence of a demand for this type of conscious control from the external environment such as might be provided by exposure to written language in the context of learning to read.

The sequence appears to play out at different points for different aspects of language (e.g. phonemes, morphemes, syllables, rimes, words) [5], with what is known about the development of phoneme and morpheme awareness reviewed below. For the most part, studies of the English language will be reviewed initially and then the critical issue of cross-linguistic variation will be addressed separately.

## Phoneme Awareness

### Development of Phoneme Awareness

Language development is thought to depend on implicit representations of the phonology or sound of words, initially stored as unanalysed wholes at the lexical level. This information is successively restructured during childhood to incorporate the increasing level of sub-lexical detail necessary to discriminate among a growing vocabulary of phonologically similar words [6, 7]. Several authors have suggested that this speech perception system underpins early sensitivity to speech sounds, such that children will show sensitivity to larger sounds like syllables and rhyming sounds before displaying sensitivity to phonemes after lexical representations have been organised segmentally [8, 9].

Influential work in the 1960s and 1970s demonstrated that preschool children were indeed unable to perform explicit phoneme segmentation tasks such as deleting or tapping out the phonemes within simple monosyllabic words (e.g. *mat*), although performance was observed to improve by school age [10, 11]. This contrasted with a more developed ability to tap out the syllables in multi-syllabic words (e.g. *cucumber*), evident even during the preschool phase [11]. The inaccessibility of phonemes to conscious control prior to school age has subsequently been replicated widely across a number of awareness tasks requiring phoneme manipulation (see [12] for a review).

In an attempt to assess more implicit levels of sensitivity to phonemes during the preschool period, speech perception tasks that do not require meta-linguistic control have been employed. These tasks have uncovered some sensitivity to phonemes among preschool children, although performance falls below the level of responses to larger sounds and may be best for phonemes located initially in words [13]. For example, in judging whether spoken word-pairs sounded similar, all 5-year-old preschoolers tested achieved six consecutive correct responses with shared syllables (e.g. hammer-hammock) but only 25% of the group achieved this criterion with shared phonemes (e.g. steak-sponge) [14]. Kindergartners were found to use global similarity rather than phonemic similarity to categorise syllables in this type of syllable similarity task, in contrast to adults who rely on phonemic similarity [15].

Therefore, an established literature supports a pattern of increasing awareness of phonemes during the preschool to early school period. Phoneme awareness is initially rather implicit and uncovered using tasks that assess sound similarity but, after school entry, children display a growing ability to manipulate and reflect on the phonemic sounds in spoken language.

### Links Between Phoneme Awareness and Reading

Awareness of several speech sounds has been examined in relation to reading development. Much has been written about a possible role for an early awareness of rhymes in beginning reading [12] since preschool children have been observed to perform well when rhyming words are used in awareness tasks like oddity and same-different matching [13, 14]. Although early rhyme awareness correlates weakly with later phoneme awareness and a persistent insensitivity to rhyme may pattern with other phonological problems in reading difficulties [16], investigations have shown that early rhyme awareness does not make a significant independent contribution to subsequent word reading [17, 18••].

Interestingly, when rhyming skills are assessed using awareness tasks that demand more meta-linguistic control or manipulation, young children score very poorly, even those children with excellent (implicit) rhyme matching and rhyme oddity performance [5]. Such findings highlight the importance of two factors in assessing links between meta-linguistic awareness and reading: (a) considering task demands, in particular the level of meta-linguistic control required, and (b) using the same task when comparing awareness of different units (like rhymes and phonemes). The basis of the error in inferring a role for rhyme awareness in beginning reading was the level of awareness tested. An implicit level of awareness of rhyme does not appear to be sufficient to allow the manipulation of sub-lexical units of sound that is required to decode new words.

Empirical evidence from longitudinal and training studies indicates instead that explicit awareness of phonemes plays an important and causal role in learning to read [19••]. Learning to read itself seems also to stimulate the development of phoneme awareness, such that a reciprocal relationship exists between these skills [20]. Alphabetic letters and their sounds have long been seen as providing a concrete realisation of the phonemes in speech, which may help to stimulate the development of sensitivity to phonemes, especially word initial phonemes [21, 22]. This may be necessary given the evidence from speech perception research [23] showing that phonemes are deeply embedded in the speech stream and that speech consists of co-articulated gestures rather than perceptually discrete phonemic units [24]. A cognitive effort seems to be required to extract these phonemes as attention in language use is focused on the meaning of speech utterances rather than on the phonemic components of words; indeed, explicit phoneme awareness does not appear to develop unless required in the course of an activity such as learning to read [3••, 4].

A further qualification to this is that it may be the type of instruction that is specific to learning to read an *alphabetic* orthography that is important for the development of explicit phoneme awareness. Alphabetic orthographies like English code the phonemes of spoken language using letters or letter-groups (graphemes) and reading instruction that emphasises these grapheme-phoneme relationships may be required for meta-linguistic awareness of phonemes to emerge. The letter-sound knowledge and word decoding exercises typical of *phonics* reading instruction has been shown to provide the necessary external demand for this to happen [25••].

Thus, reading acquisition and phoneme awareness appear to be related reciprocally. Brain imaging data confirms this dynamic relationship with literacy acquisition enhancing the activation of areas of the brain involved in speech processing (e.g. planum temporale, visual word form area), and changing white matter organisation associated with phoneme awareness and grapheme-phoneme decoding in the left arcuate fasciculus [3••]. One result of this is that after learning to read, the way in which phoneme awareness tasks are performed appears to change, with performance becoming subject to the influence of spelling knowledge [19, 26].

### Cross-Language Variation

Heterogeneity in the characteristics of spoken and written languages creates a need for adaptive models of reading development that acknowledge variation in the involvement of phonology in the acquisition process across languages. Phoneme awareness is recognised to have most relevance for learning to read orthographies in which the written symbols code phonemes. The psycholinguistic grain size model [27, 28••] attempts to incorporate variation in learning to read such alphabetic orthographies by highlighting three challenges faced by children during the acquisition process: (a) *availability*—the speech sounds that a child is (explicitly) aware of at the beginning of reading acquisition; (b) *consistency*—the regularity of the relationships between spelling and sound in the orthography; and (c) *granularity*—the grain size of the written units that represent spoken sounds determines whether a large or small number of units have to be learned .

The availability question has most relevance for the current review and was addressed directly in a longitudinal comparison of phonological and reading development in six alphabetic orthographies (English, French, Greek, Icelandic, Portuguese, and Spanish) [25••]. Considerable variation in explicit awareness of sound was observed at the beginning of the first year of reading instruction, which was associated with spoken language characteristics (e.g. linguistic rhythm) and preliteracy skills. For explicit awareness of *phonemes*, the English and Icelandic groups produced higher scores than the French, Greek and Portuguese groups. The hypothesis that vocabulary was the driving force behind the emergence of phoneme awareness [6, 7] was not supported by this outcome since the high-performing English-speaking children were a year younger than the other language groups.

After the first year of reading instruction, a dramatic improvement in explicit awareness of phonemes was apparent across all the language groups. Although the children had faced a similar granularity challenge, the consistency of the relationship between the graphemes and phonemes in the languages had varied substantially from regular (transparent) in Spanish and Greek to highly irregular (opaque) in English. Nevertheless, all groups showed ceiling-level explicit phoneme awareness after their first year of learning to read.

This finding was attributed to the influence of the phonics reading instruction experienced by all of the language groups, in which letter sounds were taught together with simple exercises to practise grapheme-phoneme decoding skills. A comparison of two matched groups of French-speaking children tested this hypothesis experimentally. The groups received either phonics instruction or whole-word instruction during the first year of learning to read. The outcome confirmed this explanation since the groups differed significantly in explicit phoneme awareness at the end of the first school year, with only the phonics instruction group achieving ceiling-level explicit phoneme awareness [25••].

Beyond the first year of acquisition, explicit phoneme awareness predicts early reading speed and accuracy across a range of alphabetic orthographies; however, the predictive power of this relationship strengthens as consistency decreases and orthographic complexity increases [29].

The importance of studying language and reading development across languages can be seen in the insights that this affords into the factors that govern performance. The cross-language similarities and differences have led to a greater understanding of the development of phoneme awareness in alphabetic orthographies.

### Phoneme Awareness in Developmental Dyslexia

Developmental dyslexia is associated with a phonological deficit where phonemic impairments cluster with other phonological difficulties to produce inaccurate and/or slow decoding [30•]. The phonological deficit has the highest prevalence in dyslexia [31•, 32] and explains substantial variance in reading [18••, 32]. Further, interventions which combine phoneme awareness training with phonics reading instruction have a positive and sustained impact on dyslexic reading [33].

Cross-linguistic work indicates that explicit phoneme awareness is a concurrent predictor of developmental dyslexia across alphabetic orthographies, most particularly when the level of orthographic complexity is high [34••]. This universal pattern of difficulty is reflected in neuroimaging reports of dyslexic disparities within the left dorsal pathway (e.g. arcuate fasciculus), associated with phonological processing [35].

## Morpheme Awareness

### Development of Morpheme Awareness

Early in language development, children join morphemes together spontaneously to create new words to fill gaps in their vocabulary. The formation of words arises from three main linguistic systems of combining morphemes: (a) inflectional morphology, which changes the grammatical function of a word to encode information such as tense and number without altering the word class; (b) derivational morphology, which alters the meaning of a word and may change the word class; and (c) compounding, which creates new meaning by combining free morphemes. Compounding appears to be the most accessible word formation process for young children due to its simple structure and semantic transparency, with affix usage appearing later in development under the influence of type frequency and productivity [36].

In studies which have investigated this ability more formally using morpheme awareness tasks, the same questions about implicit and explicit processing arise as were discussed in relation to phoneme awareness. An additional issue is that studies of morpheme awareness sometimes use written tasks rather than the purely oral tasks typically of the phoneme awareness literature e.g. [37]. This is a critical factor to consider in reviewing this literature and, to distinguish morpheme awareness from reading skill, only studies using oral tasks will be reviewed in this section.

The formative study in this field assessed English-speaking preschoolers aged 4.5 years on their ability to use inflected and derived forms to complete sentences. Better performance was observed for inflected than derived items, especially for the progressive tense and for plurals (e.g. This is a man who knows how to zib. What is he doing? He is (*zibbing*)) [38]. This task required production of inflexions and derivations to order and was seen as more demanding of conscious control than the spontaneous productions by children to fill lexical gaps in everyday communication. This question of conscious control was studied directly in a comparison of the explicit type of production required by sentence completion tasks with performance in a more implicit judgement task (e.g. A person who teaches is a teacher? (*yes/no*)) [38]. Results confirmed that 6.5-year-old first graders found inflectional morphology easier to manipulate than derivational morphology in the explicit task. Further derivational items with phonologically transparent relationships between the root and derived form (i.e. quick-quickly) were easier to produce than those with opaque relationships (i.e. long-length). When performance in the implicit and explicit tasks was contrasted using the transparent derivational items, higher scores emerged in the more implicit judgement task, although this task only assessed the very familiar agentive and instrumental forms of the suffix -er.

Non-lexical items can also be used to form new derivations in production tasks (e.g. Someone who lums is a ? (*lummer*)), which creates a higher demand for abstract knowledge about the rules governing morpheme combination since lexical knowledge alone cannot provide the answer. This increases the difficulty of the explicit task further with accuracy developing gradually across early schooling [39, 40•]. Similar developmental trajectories have been reported using other explicit tasks (e.g. analogy [41]) (see [42] for a review).

In this literature, there has been a tendency for more implicit tasks, inflectional morphology and a small number of frequent suffixes to be studied with younger children, whereas more explicit tasks, derivational morphology and a wider range of suffixes have been used with older children. These methodological differences have hampered comparison of morphological development across age levels and suffix types. However, the growing number of published findings appear to be converging on the following points: (a) differing levels of morpheme awareness can be distinguished using implicit and explicit tasks, (b) awareness of inflectional morphology emerges prior to awareness of derivational morphology, (c) explicit morpheme awareness develops gradually during schooling rather than being coincident with the onset of reading instruction, and (d) morpheme productivity, frequency and phonological transparency influence the acquisition process.

### Links Between Morpheme Awareness and Reading

The well-established ink between phoneme awareness and word reading described above modifies the question to be posed in this section. The question becomes whether morpheme awareness makes a contribution to reading that is *additional* to and independent of the contribution made by phoneme awareness, at least for alphabetic orthographies. Controlling for phoneme awareness is especially important given that morphemic relationships which lack phonological transparency are a known source of difficulty in morpheme awareness tasks [43], and may require good phonological skills to resolve [44].

Investigations of morpheme awareness and reading that control for phoneme awareness show that explicit (inflectional and derivational) morpheme awareness contributes a small yet significant amount of variance to concurrent grade 1 word reading [43] but not to later grade 3 word reading [45•]. Grade 2 explicit (inflectional and derivational) morpheme awareness predicts independent longitudinal variance in grade 3, 4 and 5 word and pseudo-word reading [45•, 46], although concurrently measured morpheme awareness was shown to be a stronger predictor of grade 3 reading abilities [45•]. Training studies together with the longitudinal relationships described here strongly suggest that the effect of morpheme awareness on decoding is causal [47••].

Converging evidence suggests that root words are pivotal to this relationship as it develops. Embedded word masked priming has been observed across grades 2 to 5 in lexical decision, facilitating the performance of good readers [48•]. In grades 3 and 5, reading accuracy for derived words was observed to be influenced by root word frequency even after controls for family size, family frequency, surface frequency, semantic relatedness and neighbourhood size [49•]. Moreover, morpheme awareness and root word reading were predictive components in a model explaining the polysyllabic polymorphemic word reading ability of readers in grades 3 and 4, more so than non-morphological (syllabic- or phoneme-based) knowledge [50].

One advantage of developing an awareness of the morphological structure of words is that it can help children to understand new complex words that they encounter in text; indeed vocabulary growth in later childhood is thought to benefit from this type of morphological analysis [51]. The automatic activation of links between sub-lexical orthographic patterns and meaning appears to start early since 7-year olds show interference from the semantic properties of embedded words in semantic competition paradigms [52]. Morphological analysis has been observed to make a significant contribution to reading comprehension alongside more traditional measures of morpheme awareness [53, 54]. An exploration of grade 3 children’s performance on these variables using multivariate path analysis, revealed that as well as a strong direct path between morpheme awareness and reading comprehension, there were two indirect paths: one via morphological decoding and word reading, and the other via morphological analysis [55••].

Altogether, this evidence supports a view of reading development in which repeated exposure to orthographic morphemes leads children to become aware of morphological structure, with the result that morphemic units become consolidated during schooling and increasingly important in the reading of complex words [56]. The preliminary indications are that the associations identified between morpheme awareness and reading constitute a reciprocal relationship [57•]. Finally, the emergence of explicit morpheme awareness appears less heavily dependent than explicit phoneme awareness on the external demand imposed by reading instruction, although the impact of school instruction about morphology awaits detailed investigation.

### Cross-Language Variation

The responsiveness of morpheme awareness to the influence of frequency, productivity and phonological transparency during development implies that the linguistic characteristics of the spoken language constrain the acquisition process. Systematic investigations of morphological development are now appearing in a wide range of languages [58], and cross-linguistic studies that contrast development in these different languages are contributing to understanding of the extent to which developmental variation occurs (see [59] for a review).

English shares some of the Latinate morphology typical of Romance languages like French due to historical influences. However, the Germanic origin of English means that the Latinate morphological system is less prevalent and less productive than in French. For example, although both languages use derivational morphology, word formation via compounding is used much more frequently in English than in French. A further point is that the phonological transparency of derivational morphology is reduced in English due to the lack of stress on affixes (cf. French). Therefore, these language characteristics predict a greater salience for derivational morphology in French than in English. This prediction appears to be borne out by experimental findings showing earlier sensitivity to derivational morphemes than is typical of English speakers, including explicit awareness of prefixes and suffixes and the production of non-lexical derivations from pseudo-word bases in kindergarten [39], and better reading of suffixed than pseudo-suffixed pseudo-words by grade 2 [60•].

This possibility was investigated directly in a cross-sectional comparison of French- versus English-speakers’ explicit awareness of derivational morphology between grades 1 and 3 [40•]. The results were consistent with accelerated development of knowledge about derivational morphology among the French speakers regardless of whether the language groups were matched on schooling or age (with vocabulary controlled). The French children knew a wider range of suffixes and were more able to generalise this knowledge to produce novel derivations in a production task. It seems possible that these morpheme awareness skills translate into the more pronounced effects of morphological processing observed in the visual word recognition of young French readers compared to their English-speaking counterparts [61•].

By contrasting English with Chinese, a different picture emerges since word formation via compounding is more prevalent in Chinese than English. Superior performance in a compound analogy task was found in Chinese across grades 2 to 6 [62]. It should be noted, however, that as this was not a purely oral awareness task, the influence of Chinese orthographic knowledge must be taken into account. While morphemic boundaries are not marked by orthographic symbols in most alphabetic orthographies, the coincidence of Chinese logographs with spoken syllables and morphemes may serve to increase the salience of morphemes. Benefits from preliterate awareness of both syllables and morphemes in shaping post-literate morpheme awareness and the recognition and understanding of Chinese characters and text have been observed over an 8-year period [63•]. Indeed, the wider literature on Chinese shows that morpheme awareness as assessed by compounding tasks has a significant association with early word reading is related longitudinally to reading development and enhances word reading when trained (see [64••] for a review).

While further cross-language research is necessary to establish the mechanisms underlying the relationship between morpheme awareness and reading, the outcome to date appears consistent with the influence of the following factors on developmental variation: (1) the prevalence of different morphological systems, (2) the productivity of these systems, (3) the phonological transparency of morphemes in the spoken language, and (4) the orthographic transparency of morphemes in the written language.

### Morpheme Awareness in Developmental Dyslexia

In alphabetic orthographies, the involvement of morpheme awareness in developmental dyslexia is still under investigation. Broadly, dyslexics tend to show reading-level appropriate morpheme awareness, an argument against morpheme awareness being a causal factor in dyslexic difficulties [65••]. Where dyslexics do perform poorly in morphemic tasks, this may simply be due to a lack of *phonological* transparency in the morphological relationships assessed [44]. Indeed for university students with developmental dyslexia, morpheme awareness appears to offer an important compensatory mechanism for phonological difficulties [66•, 67].

The picture may be different for developmental dyslexics reading morphographic orthographies like Chinese [64••], since morpheme awareness emerges as a reliable predictor of risk for reading difficulty [68•] and neuroimaging indicates disruption to the left ventral pathway (e.g. inferior longitudinal fasciculus), associated with morphological processing [69]. Nevertheless, the suggestion that morphemic deficits play a causal role in dyslexia in Chinese remains controversial. This is due to the widespread confounding of morphemic and reading skills in assessing morpheme awareness, and evidence that dyslexics show morphological processing lower than expected for age but appropriate for reading level, as in alphabetic orthographies [65••].

## Conclusions

A universal model of reading development is necessary to account for the influence of phoneme and morpheme awareness on the typical and atypical development of word reading and reading comprehension across the global range of spoken and written languages. The review presented above suggests that both aspects of meta-linguistic awareness can be reciprocally related to learning to read but that the relative importance of each may be subject to cross-linguistic variation. The lexical quality hypothesis [70, 71] captures the dependence of reading development on the quality of the representation of phonology, orthography and meaning. This may form a useful framework for a universal approach to modelling reading development that is responsive to statistical learning based on individual abilities and experience during the acquisition of spoken and written language [72••, 73].
